# The Impact of Age Stereotypes and Age Norms on Employees’ Retirement Choices: A Neglected Aspect of Research on Extended Working Lives

**DOI:** 10.3389/fsoc.2021.686645

**Published:** 2021-06-01

**Authors:** Sarah Vickerstaff, Mariska Van der Horst

**Affiliations:** ^1^ School of Social Policy, Sociology and Social Research, University of Kent, Canterbury, United Kingdom; ^2^ Department of Sociology, VU Amsterdam, Amsterdam, Netherlands

**Keywords:** ageism, age stereotypes, age norms, older workers, extending working lives, qualitative interviews

## Abstract

This article examines how older workers employ internalized age norms and perceptions when thinking about extending their working lives or retirement timing. It draws on semi-structured interviews with employees (*n* = 104) and line managers, human resource managers and occupational health specialists (*n* = 52) from four organisations in the United Kingdom. Previous research has demonstrated discrimination against older workers but this is a limiting view of the impact that ageism may have in the work setting. Individuals are likely to internalize age norms as older people have lived in social contexts in which negative images of what it means to be “old” are prevalent. These age perceptions are frequently normalized (taken for granted) in organisations and condition how people are managed and crucially *how they manage themselves*. How older workers and managers think and talk about age is another dynamic feature of decision making about retirement with implications for extending working lives. Amongst our respondents it was widely assumed that older age would come with worse health—what is more generally called the decline narrative - which served both as a motivation for individuals to leave employment to maximize enjoyment of their remaining years in good health as well as a motivation for some other individuals to stay employed in order to prevent health problems that might occur from an inactive retirement. Age norms also told some employees they were now “too old” for their job, to change job, for training and/or promotion and that they should leave that “to the younger ones”—what we call a sense of intergenerational disentitlement. The implications of these processes for the extending working lives agenda are discussed.

## Introduction

In this article we address how age relations in organisations impact on the willingness of older workers to extend their working lives. Internationally, an important policy phrase has been “live longer, work longer” (OECD, 2006; Street and Ní Léime, 2020). Policymakers are trying to stimulate older people to extend their working lives, for example in the context of the United Kingdom (UK) by abolishing mandatory retirement ages, increasing the State Pension Age, and by introducing age discrimination legislation (see e.g., ILC-UK, 2017, and Lain, 2016, for an overview). These policies are introduced in response to predicted increased population aging and worries about increasing dependency ratios and the affordability of welfare states. There are various problems with this policy narrative as well as its proposed solutions, including that it appears to be a “one-size-fits-all” approach that ignores the different realities of various groups of older workers (for more detail see e.g., Street and Ní Léime, 2020). Another issue is that such policy changes occur in social contexts of considerable ageism.

Ageism is commonplace and embedded at all levels: in public policy narratives when talking about older workers, in popular narratives about baby boomers stealing prosperity from younger generations; in organisational regimes which favor the ideal fit and healthy worker (aka not “the old”) and in workplace banter about older workers being put out to pasture. Although there is a long history of research that shows that negative images of older workers are related to discrimination against these employees (see e.g., Chiu et al., 2001; Macnicol, 2006; Hurd Clarke and Korotchenko 2016; Earl et al., 2018), there is less attention to how older workers may themselves make labor market decisions based on internalization of these narratives. Recent reviews have asked for more qualitative research on ageism (Harris et al., 2018) and we seek to begin to address this gap in the literature.

## Theoretical Considerations

We are seeking to extend our understanding of various components that are part of ageism. Ageism involves active discrimination, but also stereotyping and age norms. The latter two may operate against people as well as being internalized by those subject to them. It is typical in organisational studies to research ageism as perpetrated by managers against employees (for example, Chui et al., 2001; Henkens, 2005; for an overview of the workplace literature see Naegele et al., 2018). Whilst there is evidence for discriminatory behavior by managers against older (and younger) employees this is a limiting view of the impact that ageism may have in the work setting.

Conceptually we see “age” “as a socially and culturally constructed category” (Krekula et al., 2018, p.37; see also Calasanti and Slevin, 2001; Calasanti, 2020). Regarding older workers, we need to understand how age is constructed and performed in the workplace. Age stereotypes identify what is routinely attributed to particular age groups. Prevalent stereotypes about older workers include that they are “(a) less motivated, (b) generally less willing to participate in training and career development, (c) more resistant and less willing to change, (d) less trusting, (e) less healthy, and (f) more vulnerable to work-family imbalance” (Ng and Feldman, 2012, p. 821; see also Posthuma and Campion, 2009). In their meta-analysis, Ng and Feldman (2012) only found some evidence for (b), though this does not say *why* they would be less willing to participate in training and career development. Hurd Clarke and Korotchenko (2016) summarize existing literature as follows: “the research suggests that ageism is often deeply internalized as individuals accept stereotypes that depict later life as a time of poor health, cognitive impairment, dependence, lack of productivity and social disengagement” (p. 1759). Part of this is an internalized health-decline-narrative, which has been referred to as “health pessimism” (see e.g., Brown and Vickerstaff, 2011). It has been claimed that because workers themselves believe the stereotypes, many cases of age discrimination go unnoticed (Laczko and Phillipson, 1990). Recent research suggests that stereotypes about motivation, mental and physical health remain very persistent (Kleissner and Jahn, 2020) and age and health perceptions might also have an impact on older workers’ motivations to continue or leave work (Van der Horst, 2019).

Next to age stereotypes, age norms (at which age should you do what?) are also important to take into account. In an employment context, ageist ideas will play out in interpersonal interactions but also institutionally through policies and routine practices (Martin et al., 2014; see also Krekula (2009) on age coding practices). Age norms are frequently normalized (taken for granted) in organisations and condition how people are managed and how they manage themselves. Age norms are related to how people manage themselves because they will inform people’s understanding of their own age and its implications in the work context. Ageism exists through social relations rather than primarily being a characteristic of individual behavior (cf. Van der Horst and Vickerstaff, 2021:4), which is exemplified by the fact that: “older workers” are only “old” in relation to other presumably “younger workers” and vice versa. The rise of narratives about intergenerational fairness (see Willetts, 2010; Wildman et al., 2021) may feed into concerns about older workers job blocking younger generations. This may in turn have increased the impact of age norms on labor market considerations in recent years.

Few studies have specifically researched the impact of internalized ageism on older workers but some studies do refer to cases of self-exclusion or what Romaioli and Contarello (2019) in a different context have referred to as a self-sabotage narrative: being “too old for”. Minichiello et al. (2000) show with an Australian sample that “older people may adjust their lives so as to accommodate problems they encounter” and that “older people may simply “drop things out of their life” once access becomes difficult rather than lobby for improved resources” (p. 263), Gaillard and Desmette (2010) showed using a Belgian sample that positive stereotypes of older workers were related to lower early retirement intentions and a higher motivation to learn and develop, and in 2008 that identifying as an “older worker” was related to higher early retirement intentions (Desmette and Gaillard, 2008). Brown and Vickerstaff (2011) suggested that health pessimism may be a factor in retirement planning.

The main aim of this article is a qualitative exploration of the role of internalized age stereotypes and norms in employment decisions of older employees in the United Kingdom. As much is already known about *which* stereotypes exist, we focus more on how older workers and their managers deploy these stereotypes and age norms when talking about their working lives; we are interested in the social relations of age; how ageism is performed and reproduced through interactions and how this affects thinking about retirement.

## Data and Method

This article is based on individual semi-structured face-to-face employee interviews (*n* = 104), as well as interviews with line managers, human resource and occupational health managers (*n* = 52) divided over four organizations. The organizations were located in different sectors, with varying workforces, and in different regions in the United Kingdom (the South East, North West, West, Wales and the Home Counties; for further details see Table 1). Interviewees were selected out of employees aged 50 or over who volunteered to participate using a maximum variation sampling strategy (Patton, 1990; Flyvbjerg, 2016). Managers were selected because they had responsibilities for workforces which included some older workers. In this article we concentrate primarily on the interviews with employees. The data were collected between 2014 and 2016 and interviews were held at the work location during working hours, but in a setting that ensured confidentiality. The average length of interview was between 45 and 50 min, they were digitally recorded, and transcribed verbatim.

**TABLE 1:** Number of participants by case study organization and employee details.

organization	Hospitality	Local government	Transport	Manufacturing
Type of respondent				
Human resource/pension/occupational health managers	3	5	6	13
Line managers	5	9	6	5
Employees	22	37	19	26
Employee demographics				
Gender				
Female	64%	54%	37%	19%
Male	36%	46%	63%	81%
Age				
50–59	64%	76%	74%	42%
60–64	32%	24%	21%	19%
65+	5%	–	5%	4%
50+ (specific age undisclosed)	–	–	–	35%

Percentages may add up to more than 100% due to rounding.

Employees were interviewed about their retirement plans, experiences of age discrimination at work and their views on policy changes around extending working lives. Questions were open ended encouraging respondents to articulate issues salient to them. Interviews with managers centered on how their organisations managed older workers. The focus in this paper is an analysis of how people talk, the language used, about age and ageing. Though the focus of the interviews was not on internalized age norms and how this affected work decisions, these topics emerged in many interviews when people gave their views on changes in policies, experiences at work, and/or their plans for the future. It may be that the data contains many examples of internalized age-stereotypes because it was not directly questioned. Spedale (2018) notes in her study how the identification as “an older worker was predominantly unconscious and informed by age-related hidden assumptions and taken-for-granted beliefs” (p. 41). By identifying age norms and stereotypes when talking about different topics, the data may contain a more “natural” discussion of age at work. The qualitative data were analyzed thematically. An initial deductive coding frame was developed based on the larger project’s research aims and empirical and theoretical interests. In addition, an inductive open coding approach was taken so that themes and issues could arise from the data. After identifying internalized ageism as an emerging theme, the interviews were thematically recoded in NVivo 12 using the framework for analysis in Table 2 and read and reread for comments on the relationship between ageism and employment decisions (on framework analysis see Ritchie et al., 2003).

**TABLE 2:** Framework for qualitative analysis.

	Stage 1	Stage 2	Stage 3	Stage 4
Rq: To what degree do older workers accounts involve internalized negative stereotypes about issues such as memory, cognitive and physical decline and positive stereotypes about warmth and dependability?	Read transcripts for positive and negative stereotypes and use of language	Code for: 1) memory issues, physical capability, productivity, attitudes toward training and development and IT, dependability, expertize, knowledge, warmth. Deductively and inductively 2) for language used	Review codes, identify related codes/relationships between codes Identify any common or particular language forms	Develop conceptual categories

Our approach here is part of the discursive turn in gerontology (Previtali et al., 2020). Through our focus on talk we hope to both expose the ageist narratives in society and in organizations but also to explore how people actively construct their own understandings of reality. Our purpose is not to attempt to replace other explanations of the dynamics of retirement decisions but rather to add another layer to our understanding. The direct quotations from interviews below are selected as indicative for the identified category. Employee interviewees are identified by gender and age; managerial employees by their role.

## Results

### Context

In all of the organizations the overwhelming majority of employees said there was little direct age discrimination for example in access to training. There were some individuals who felt they had been passed over for opportunities or targeted for redundancy because of their age, but in general employees agreed with managers that their organizations were not overtly ageist.

I’ve not come across any discrimination, other than the banter around your desk kind of thing, you know. (Male, 57).

I get as much abuse as I think we dish out, the old fart in the office, but no I don’t see any different treatment. (Male, age undisclosed).

As these two quotes demonstrate people think of ageism as about direct discrimination and do not see that age stereotypes and norms are embedded in everyday interactions. Both managers and employees regularly employed ageist stereotypes when talking about older workers or about themselves, what we might refer to as casual or normalized ageism. These reflected the standard negative stereotypes about memory issues, physical capability, productivity, attitudes toward training and development and IT:

I’m just cast as a scatty old lady, you know. (Female, 52).

Although people live longer they don’t necessarily—it’s hard to predict at what point they’re no longer going to be really capable of doing their job, to be blunt. (Male, 56).

I suppose might be that some of the older people might be more kind of dinosaurs in terms of technology and slower to pick up the latest, you know, electronic tools and things. (Male, age undisclosed).

There were also more positive stereotypes about dependability, expertise, knowledge, warmth:

I think as you get older you get more of a sense of responsibility. You don’t like letting people down. You tend to work your way round problems rather than think, oh no, I’m not doing this, I’ll go somewhere else. (Female, 61).

You know, when there’s a problem they come running to us first, we’ll get it sorted. Yeah, I suppose I think they do look at it like that, yeah. (Male, 54).

Managers made explicit comparisons between older and younger workers:

I think certain individuals, as they get older and more established in their role, choose not to pick up on every opportunity that’s put before them, but the excitement comes for us as managers for the younger guys who are, “Yeah, what can I do? Give us more, can I do that, can I do this?” and that keeps that process going. […] if the older guys don’t want to pick up on it, it’s not because it’s not available and we would hold it back for them, it’s definitely available but sometimes their attitude or their energy towards it is less so than the guys further down the chain. (Male, 50 interviewed as an employee but with line manager responsibilities)

The prevalence of age based stereotypes was recognized by some employees and to a degree resisted.

Now training, I think it was perceived, and I think it was a wrong perception, that these people had no experience of working on computers, which is completely wrong because those guys like everybody else were going down Tesco’s and Curry’s buying laptops and desktops and playing around on Facebook and YouTube just like everybody else. (Male, 51).

Sometimes it was not the stereotype itself that was resisted, but the degree to which it would apply to them. They considered themselves as not yet “old” as stereotypes about what it means to be “old” did not apply. Many of the employees interviewed said that they did not “feel” their chronological age and felt that they were valued but at the same time many expressed concern about how others might see them or overlook them:

you do become invisible … but it’s like you are cannon fodder in a way, you’re just there to keep the wheels turning. (Female, 57)

### Categorizing Talk About Age

Two conceptual categories developed from the analysis of how managers and employees deployed ageist stereotypes and age norms when talking about work opportunities, retirement timing and extending working lives: 1) the prevalence of a decline narrative, namely the widely held assumption that ageing inevitably brings worsening physical and cognitive health, and 2) the prevalence of an intergenerational narrative. The latter had two dimensions: one about being “too old for” something and the second related to intergenerational disentitlement; the need to step away and privilege younger workers.

Both narratives involve a comparison. The decline narrative conditions how people view the implications of getting old and has a role in how they think about continuing or ending work; here people compare themselves with an imagined future self. The intergenerational narrative is how people place themselves in relation to other generations in the workforce, here people compare themselves (and are compared) to others.

#### Decline Narrative

In discussing future retirement, the health and mortality of colleagues, family, and friends were constant topics leading to something which may be referred to as the decline narrative (Gullette, 2004) or “health pessimism” (cf. Brown and Vickerstaff, 2011). This was expressed repeatedly as not knowing when “one’s time is up” or being able to predict how long decent health would last. For many, this expected age related decline in health translated into a desire to retire in time to enjoy some leisure:

One lady, she retired, she was only retired two months and she passed away. And, you know, you think, I don’t want that to be me. And I know you can never say, but I don’t want to work my whole life just to retire and then die. I’d like to enjoy a bit of free time. (Female, 50).

There was a strong sense of not wanting “to run out of time” and instead wanting to “maximize enjoyment of their remaining years in good health” (cf. Pond et al., 2010). In relation to the raising of the state pension age in the United Kingdom some felt that policy might force people to work too long, prejudicing their ability to enjoy retirement, this was especially true for those in manual occupations.

I can understand that you shouldn’t have to retire at 65 or whatever age they want to choose, because there’s lots of people perfectly capable of working and they want to, but I do think we’re in danger of keeping people in work who are not fit, because your bodies do start to wear out a bit and the older you get the more susceptible you are to things going wrong and then what are we going to do with those people, what are they going to do? (Female, 58).

For some others the decline narrative worked the other way around and they saw work as a means for staving off the inevitable decline. Paid work was for them a way to stay active and this would be necessary to stay healthy (longer):

Inside I still feel 35 [laughs], shame that the mirror doesn’t agree with me, but [both laugh], yeah, I mean the job is very physical, so but I look on that as being like keep fit, I’m a great believer in use it or lose it, and I think if I’d have given up work at 60 I’d have been a little old lady by now, probably about three stone heavier and gray haired. (Female, 64).

Not all decisions to stop working or extend working life are related to age stereotypes; some look forward to a period in which they have time for hobbies as they are in a financial position to stop working. Others have more negative reasons to give up their job such as health problems and being unable to continue working. Again others are happy to continue working or are not financially able to retire even though they would prefer to. Next to these push and pull factors, which have been identified in previous research, our data does suggest that the decline narrative also plays a role in how people weigh up the factors encouraging or discouraging continued employment. Many employees talked about a fear of being viewed as old and used pejorative language such as “pottering about”, “being a dinosaur”, “doddery” in describing other older people or their future selves.

#### Intergenerational Narrative

A second narrative expressed by some of our interviewees is about comparisons between age-groups in the labor market. The interviewees are comparing themselves with younger workers and either consider themselves as now “too old” for certain opportunities, or younger workers more worthy for these opportunities. This comparison can be made implicitly or explicitly. The first dimension of this narrative is the “too old for” (TOF)-narrative, which is based on an implicit comparison, where the older worker now considers themselves “too old for” their job or development:

I’ve spoken to other people and they’ve said it’s a young person’s game. […] multitasking in your head and you’ve got three—, no, 20, 30, 40 tickets coming through and you’re trying to mentally keep hold of it all. […] I’m not a woman I can’t multitask (both laugh). So it would be very hard to keep on doing that. (Male, 52)

TOF was most clearly and commonly expressed in relation to training:

I just feel at 60 now, is that really too old for me to be able to, you know, go on all these courses? And there’s quite a few that they want me to do. (Female, 59).

In the TOF-narrative the younger “other” is implicit. But other times intergenerational comparisons are made more explicitly. Many believed that in straightforward competition organizations preferred younger over older workers and that once you are over 50 opportunities in the labor market diminish markedly:

I continually look online, in the papers, I look in places, but when you are 57 and there’s a 30 year old applying for the same job, they’re not going to take me, are they? They’re not. (Female, 57).

Whilst the lack of opportunities for older people was lamented there was a very strong feeling among many of the interviewees that rising state pension ages and the urge to extend working lives was bad for younger generations:

Give the young people who are out there a chance to get into work, because there’s a lot of people unemployed. And I think the longer we go on, the less chance there is for them to get into work, because there’s less people retiring. That’s how I look at it any road. That’s my point of view. (Male, 60).

I actually have a problem with people working longer cause—, guilt’s not the right word, but there are lots of young people who can’t get jobs, you know. (Male, 69).

A number of employees thought that it was right that opportunities *should* go to younger people. Age norms were internalized by older workers who expressed the view that they were now ”too old” for training and/or promotion and that they *should* leave that “to the younger ones”:

I’m not particularly after getting promoted, I’ll leave that for the younger ones. I’m just happy where I am and for me I would rather be in this kind of job. (Female, 56).

I don’t want to improve. I don’t mean I don’t want to improve. I will do what I’m doing. I want to give the chance to the young people. […] I’m very, very sorry, I am not interested. Give the chance to the young people. (Male, 54).

Older workers here are wrestling with it being unfair that older workers may be discriminated against whilst also feeling that they have less entitlement to work when younger groups are unemployed, are still building a career or have young families to support. Many people mentioned their children or grandchildren and how difficult the labor market and work was for them.

## Discussion

With an increasing call for employees to extend their working lives, it is important to explore all the factors that are likely to limit this policy goal. The research reported here focused on a hitherto neglected aspect that of the role of internalized age stereotypes and norms in inhibiting older workers. There is a rich literature on direct discrimination against older workers and to a large extent our managers and employees were thinking about this kind of prejudice in relation to ageism. Age discrimination legislation has been around long enough in the United Kingdom for managers and many employees to know that it is proscribed in law and hence when asked our respondents in the majority said that there was no different treatment based on age.

The language managers used to talk about older workers and the way those older workers framed their own thoughts about, work, extending working lives and retirement tells a rather different story. Age stereotypes were routinely employed with respect to older workers capabilities and potential. Age norms about what was appropriate for different age groups were used to talk about training and development or extending working lives. In this sense ageism was normalized in all of the organizations, taken for granted and to a large extent unexamined. Ageist language did not seem to have the power to shock in the way that overtly racist or sexist language nowadays might.

The decline narrative—that with age comes inevitable physical and cognitive deterioration—was prevalent in how employees talked about extending their working lives and/or retirement. It was a factor in their thinking about the desirability of employment as they aged. This was true for those identifying as in good health as well as those with current health issues. As in other studies many people were concerned to retire early enough to still enjoy some health in retirement (Pond et al., 2010; Brown and Vickerstaff, 2011). However, for a minority this decline narrative functioned as an incentive to stay in work as a means of maintaining social and physical activity and staving off the onset of ill-health. This latter view chimed more with the increasingly dominant public narrative of active and healthy aging: that work is good for you and keeps you physically and mentally fit (Department for Work and Pensions (DWP), 2017: 9; Moulaert and Biggs, 2012; Laliberte Rudman, 2015). In doing so of course it still takes the eventual and inevitable decline as its point of departure. The decline narrative has been discussed in the existing literature and our study confirms its ubiquity but we noted that it can play either a positive or a negative role with regard to extending one’s working life.

More distinctive were our findings about the intergenerational narrative. If we conceptualize age as social construct then it focuses attention on the relational aspects of age and how age relations are played out in specific contexts. A rather obvious statement is that older workers are only old in relation to some other younger reference group. However, we could clearly see in the comments of both managers and employees that such comparisons were very much alive in people’s minds. They were employed when they were thinking about career opportunities, training and development or the desirability of extending working lives. This was manifested in the “too old for” narrative, expressing a sense that there is a specific chronology for when things are appropriate in the working life. This is perhaps all the more remarkable in our sample as the majority were in the age category 50–59 (see Table 1), with presumably many years still in employment. This self-sabotaging narrative, as Romaioli and Contarello (2019) have characterized it, does lead to older people self-limiting. This means older workers potentially opting out of opportunities that are actually available.

The other dimension of the intergenerational narrative we have dubbed “intergenerational disentitlement”, as there was a strong element in our respondents’ comments that as older workers they were less entitled to training and development and possibly even to a job when compared to younger (potential) colleagues. Here many of our respondents were expressing a tension between a commitment to the fact that age discrimination is unfair and should be resisted whilst nevertheless worrying that by taking a promotion or staying in work they might be denying, by implication a more deserving, younger person. This sense of disentitlement could potentially be an important factor in a situation of redundancies, where both managers and employees may feel that if anyone should go it should be the older workers.

This sense of age-based disqualification for job opportunities might undermine formally equitable processes in the workplace; everyone may be entitled to apply for a job, a redeployment or a promotion but some older workers may define themselves as “too old” or think it should “be left for the younger ones”. Age management policies tend to focus on direct discrimination and formal equality but may do little to tackle underlying and normalized ageism of the sort uncovered here.

Individual decisions about whether to carry on working or retire are as we know complex and constrained. The interaction of health, wealth, marital status, employer action and government policy combine to structure what is possible and what is desirable (Vickerstaff, 2006; Loretto and Vickerstaff, 2012; Hasselhorn and Apt, 2015; Lain, 2016; Phillipson et al., 2019). The study reported here seeks to include in the list of dynamic variables in retirement decision making, a full and rounded sense of the impact of ageism. It has added another layer to our understanding. The power of ageism to influence end of working life actions is not limited to direct discrimination, although this still certainly plays a significant role, it also encompasses normalized and taken for granted assumptions about age norms, what is suitable for different age groups and why, as well as internalized stereotypes about older workers abilities and aptitudes.

### Limitations of the Current Research and Suggestions for Future Research

We have to acknowledge a note of caution about the generalizability of our findings. By the standards of much qualitative work we had a quite large and diverse data set. Our employee respondents covered a good spread of occupational levels in diverse organizations and the gender balance of the sample reflected the gender composition of the different organizations, with a slight over-representation of female respondents. With the weight and depth of interview material we were able to triangulate responses and have concentrated on oft repeated themes and tropes. The sample was however ethnically homogeneous with the overwhelming majority of our respondents identifying as white British. A more diverse sample including a range of the black and minority ethnic populations in the United Kingdom might have confirmed our findings or uncovered different ways of talking about age and generations. In this article we have not examined the gender differences in ageist talk but rather concentrated on the expressions and themes common to both genders. Further research could usefully delve into the subtle differences in how women and men talk about and experience age.

Our respondents were also interviewed in a particular time and place. We do not seek to diminish the importance of public policy and organizational contexts in setting parameters for what is possible for older workers. It would be interesting to see similar narrative analyses undertaken in different national contexts to see whether internalized ageism is as strong and has the same dimensions as identified here. It is also the case that public narratives of what is right or expected of older populations are in some flux as we shift progressively from a societal view of retirement as an earned right for a long working life to the duty on older people to carry on contributing to economic life. Individuals, with their own dispositions, life experiences and family contexts are wrestling with these changing new messages as are we as researchers. It would be interesting in further research to try to link more clearly the impact of public narratives about greedy baby boomers, intergenerational inequity and healthy aging on narratives in the workplace.

### The Main Contributions of This Research

We have addressed the spirit of this special issue by identifying a new pathway in retirement research methodologically and conceptually. In so doing we have added another layer to our understanding of the factors that are in play in disposing early retirement or later working. Although we cannot specify the weight or percentage contribution internalized ageism plays in decisions about paid work we have highlighted that it cannot be ignored as a factor. Methodologically we have demonstrated that in addition to quantitative analyses, case studies of organizational practice, and assessments of the impacts of public policy changes, we need to look at how people talk and think about age in the work setting. Embodied stereotypes and taken for granted age norms make a profound contribution to individual and organizational practices around extending working lives. Conceptually we have tried to deepen our understanding of ageism in the work place. We extended the narrow and limiting focus on discrimination against older workers to investigate other components of ageism, namely how older workers respond to age stereotypes and age norms in how they manage themselves.

## Data Availability

The dataset analysed for this study can be found in the UK Data Archive, with reference SN852868. https://beta.ukdataservice.ac.uk/datacatalogue/studies/study?id=852868.
